# Can we skip technetium-99 m sestamibi scintigraphy in pediatric primary hyperparathyroidism patients with positive neck ultrasound results?

**DOI:** 10.1007/s00247-023-05702-w

**Published:** 2023-07-13

**Authors:** Yudi He, Yanwen Luo, Siqi Jin, Ou Wang, Quan Liao, Qingli Zhu, He Liu

**Affiliations:** 1 Department of Ultrasound, Peking Union Medical College Hospital, Chinese Academy of Medical Science & Peking Union Medical College, No. 1 Shuaifuyuan Wangfujing Dongcheng District, Beijing, 100730 China; 2 Key Laboratory of Endocrinology, Department of Endocrinology, National Commission of Health, Peking Union Medical College Hospital, Chinese Academy of Medical Science & Peking Union Medical College, No. 1 Shuaifuyuan Wangfujing Dongcheng District, Beijing, 100730 China; 3 Department of General Surgery, Peking Union Medical College Hospital, Chinese Academy of Medical Science & Peking Union Medical College, No. 1 Shuaifuyuan Wangfujing Dongcheng District, Beijing, 100730 China

**Keywords:** Children, Primary hyperparathyroidism, Technetium-99 m sestamibi, Ultrasound

## Abstract

**Background:**

Parathyroidectomy is the only curative treatment for primary hyperparathyroidism (PHPT). Ultrasound (US) and technetium-99 m sestamibi (^99m^Tc-MIBI) scintigraphy are recommended as the first-line localization imaging modalities for PHPT in adults, but the value of preoperative imaging in pediatric patients has not been reported.

**Objective:**

To evaluate the added value of ^99m^Tc-MIBI scintigraphy in pediatric PHPT patients with positive ultrasound results.

**Materials and methods:**

Pediatric patients (≤18 years old) who were diagnosed with PHPT and underwent surgical treatment in Peking Union Medical College Hospital between January 2003 and January 2021 were included in this study. Demographic and clinical characteristics, preoperative localization US, ^99m^Tc-MIBI scintigraphy and pathology results were collected. Preoperative localization results were evaluated by comparison with surgical and pathological findings.

**Results:**

There were 32 pediatric PHPT patients with median age of 14.7 ± 2.5 years who all proved to have single-gland disease without ectopic lesions. The median lesion size was 2.85 cm (range 1.0–5.8 cm). All patients underwent US and ^99m^Tc-MIBI scintigraphy. Neck US demonstrated 100% sensitivity. Of 32 patients with a positive US, ^99m^Tc-MIBI scintigraphy was concordant in 30 (93.8%). In 2 patients (6.3%), US reported suspected multigland disease, which was correctly diagnosed by ^99m^Tc-MIBI scintigraphy as single lesions.

**Conclusion:**

In pediatric PHPT patients, US achieved high sensitivity for preoperative localization. ^99m^Tc-MIBI scintigraphy for pediatric patients with positive US results would not increase the sensitivity. Implementation of ^99m^Tc-MIBI scintigraphy could increase the specificity in pediatric patients with multigland disease suspected by US.

**Graphical abstract:**

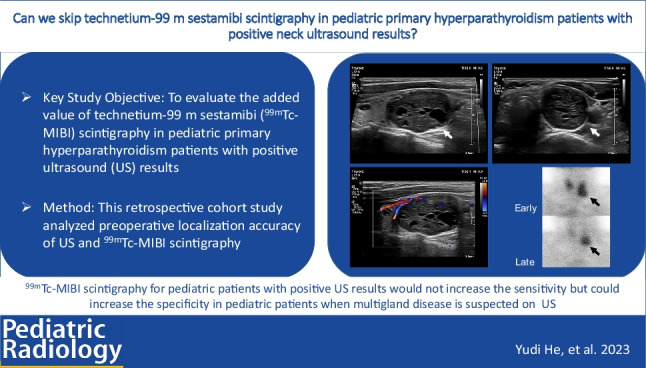

## Introduction

Primary hyperparathyroidism (PHPT) is characterized by abnormal elevation of serum calcium and parathyroid hormone (PTH) levels due to pathological parathyroid glands and involves multiple organ systems, including the skeletal, urinary and digestive systems [1]. Pediatric PHPT is less common compared to adult PHPT (incidences of 2–5/100000 and 1/1000, respectively). However, the percentage of symptomatic pediatric patients is significantly higher and treatment delays could cause a number of complications, such as nephrolithiasis, osteoporosis and bone fracture and may affect the growth and development of children [2–5].

Parathyroidectomy is the only curative treatment for PHPT [6]. The operative approach includes minimally invasive parathyroidectomy/unilateral neck exploration, which is generally applied to unilateral gland disease with relatively precise localization and bilateral neck exploration, which is usually conducted in patients with bilateral multiple gland disease, obscure localization of lesions or hereditary etiologies [7]. Therefore, preoperative localization of lesions is one of the crucial factors in planning the surgical approach to minimize trauma and achieve operative success.

Widely accepted first-line localization imaging examinations include neck ultrasound (US) and technetium-99 m sestamibi (^99m^Tc-MIBI) scintigraphy [6, 8–10]. The American College of Radiology Appropriateness Criteria in 2021 and the American Association of Endocrine Surgeons guidelines in 2016 recommend that the choice of imaging examination should be based on expert advice and regional imaging capabilities [6, 11]. Koewar et al. proposed a stepwise manner of imaging studies in which US could be performed routinely while ^99m^Tc-MIBI scintigraphy would be performed when negative US, failed operation or recurrence occurred [12]. Another study used a hypothetical model to build a localization modality in 513 PHPT patients (mean age 58.5 ± 14.1 years) and demonstrated that the value of ^99m^Tc-MIBI scintigraphy after positive US was limited, since added ^99m^Tc-MIBI scintigraphy could only change the operative plan made by referring to US positive results in 2.3% of patients [13]. Children and adolescents are a more sensitive population to radiation exposure, but data are near nonexistent regarding the value of ^99m^Tc-MIBI scintigraphy in pediatric PHPT patients with positive US results.

This study aims to evaluate the added value of ^99m^Tc-MIBI scintigraphy in pediatric PHPT patients who have positive US results.

## Materials and methods

This retrospective study was approved by the Institutional Review Board of Peking Union Medical College Hospital. From January 2003 to January 2021, pediatric patients (≤18 years old) who were diagnosed with PHPT according to serum calcium and PTH levels and underwent surgical treatment were enrolled [14]. All patients underwent cervical US and ^99m^Tc-MIBI scintigraphy for the purpose of localization before surgery. The exclusion criterion was patients with incomplete information on preoperative images. Demographics, clinical history and radiology reports were obtained from the electronic medical records. Data collected included age, sex, preoperative PTH, preoperative calcium, US and ^99m^Tc-MIBI scintigraphy imaging findings, pathology results and clinical follow-up.

Preoperative US was performed by Y.J. and H.L., both radiologists with at least 5 years of experience in parathyroid ultrasonography using one of two US machines (IU22 or Epiq; Philips, Amsterdam, The Netherlands) and a broadband linear array probe (L12–5 MHz). For each suspicious lesion identified, longitudinal and transverse views using grayscale and Doppler modes were obtained. The position of the lesions, lesion size, echo pattern and blood flow were recorded. The sonographic appearance of the adjacent thyroid gland was evaluated to provide a differential diagnosis between parathyroid gland lesions and nodular goiters.

Patients received intravenous injection of ^99m^Tc-MIBI (Atom Hitech Co., Ltd, Beijing, China) at a dose of 10~20 mCi according to routine clinical practice. Planar images of the neck and upper chest were obtained 20 min and 120 min after administration with a gamma camera and a pinhole collimator. Images of single photon emission computed tomography/CT (SPECT/CT) were acquired after 120 min on a 64-slice Philips Precedence device (Philips, Amsterdam, The Netherlands). SPECT was performed in a 128 × 128 matrix with a zoom factor of 1.0 and 32 projections over 360°. CT was performed with a current of 30 mAs, voltage of 120 kV and slice thickness of 3 mm. Each examination was independently reviewed by 2 experienced radiologists (Y.P. and Q.P.), subspecialists in endocrine imaging with more than 5 years of experience each.

The accuracy of preoperative localization was evaluated using operative and histological findings as the reference standards. The cervical region was divided into quadrants with a vertical line drawn along the midline of the neck and a horizontal line through the middle thyroid. The preoperative location was classified as left superior, left inferior, right superior, right inferior or ectopic and compared to actual location identifed at surgery.

Parathyroidectomy was conducted within 2 weeks of preoperative localization. Operation procedures were obtained from medical records focusing on operation approach (unilateral or bilateral) and intraoperative findings (size and position of lesions). Operative cure was defined as a postoperative calcium level that remained normal during a follow-up period of at least 6 months [6]. Histological findings (according to the World Health Organization [WHO] classification standard) provided etiological information [15].

Statistical analysis was conducted using SPSS 25.0 software (IBM Inc, Armonk, NY). Categorical variables were described as frequencies and percentages. Continuous variables were described as the mean ± standard deviation (SD) or median value and range. Sensitivity was calculated to reveal the effectiveness of multiple preoperative localization methods. A *P*-value of <0.05 was considered statistically significant.

## Results

### Demographic and clinical characteristics

Demographic and clinical characteristics are summarized in Table 1. A total of 32 PHPT patients who underwent surgical treatment with a median age of 14.7 ± 2.5 years old (range 9–18 years old) were included. Of 32 patients, 14 (43.8%) were female. Preoperative PTH and calcium levels were 926.60 ± 864.95 pg/ml and 3.03 ± 0.39 mmol/l, respectively. The median lesion size was 2.85 cm (range 1.00–5.80 cm). All 32 patients (100%) had single-gland disease. Of the 32 children in our series, 5 (15.6%) were also diagnosed with multiple endocrine neoplasia type 1 (MEN1). Most lesions (21/32, 65.6%) were parathyroid adenoma, followed by atypical adenoma (5/32, 15.6%) and hyperplasia (6/32, 18.8%). No ectopic lesions were reported. All patients achieved operative success and the curative rate was 100%. Of the five MEN1 patients, three had recurrent lesions during 1–7 years of follow-up after the initial parathyroidectomy.

**Table 1:** Demographic and clinical characteristics of pediatric primary hyperparathyroidism patients

	Value (*n*=32)
Age, years (mean ± SD)	14.7 ± 2.5
Sex	
Female	14 (43.8%)
Male	18 (56.2%)
Preoperative PTH, pg/ml (mean ± SD) (12.0–68.0)	926.60 ± 864.95
Preoperative Ca, mmol/l (mean ± SD) (2.13–2.70)	3.03 ± 0.39
Subtype	
Sporadic	27 (84.4%)
MEN1	5 (15.6%)
Operative findings	
Left sided, superior	8 (25.0%)
Left sided, inferior	10 (31.3%)
Right sided, superior	4 (12.5%)
Right sided, inferior	10 (31.3%)
Ectopic	0 (0%)
Lesion size, cm (median, range)	2.85 (1.00, 5.80)
>2 cm	24 (75.0%)
Histological findings	
Adenoma	21 (65.6%)
Atypical adenoma	5 (15.6%)
Hyperplasia	6 (18.8%)

*Ca* calcium, *MEN1* multiple endocrine neoplasia type 1, *PTH* parathyroid hormone

### Preoperative localization

**Fig. 1:**
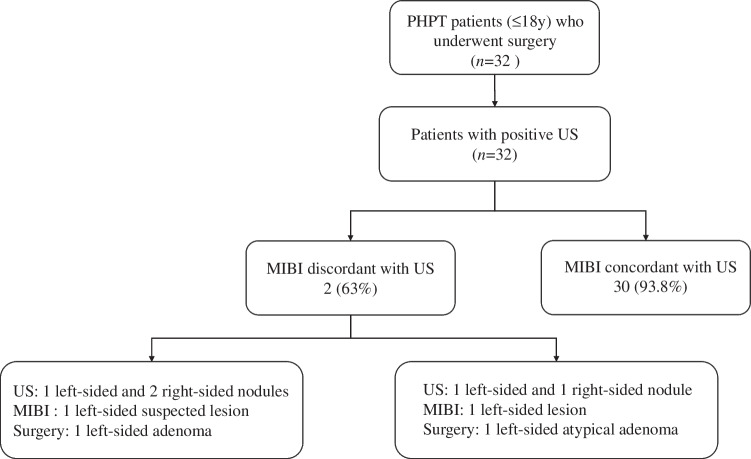
Preoperative localization in pediatric primary hyperparathyroidism patients. *MIBI* technetium-99 m sestamibi scintigraphy, *PHPT* primary hyperparathyroidism, *US* ultrasound

A positive US was defined as patients with US findings suggestive of parathyroid lesions or indeterminate nodules. Of 32 patients who underwent US and ^99m^Tc-MIBI scintigraphy, all had a positive US result, 30 patients (93.8%) had consistent findings on US and ^99m^Tc-MIBI scintigraphy and 2 (6.3%) had discordant preoperative localization (Fig. 1).

In 30 patients with concordant imaging results, surgical findings confirmed a single-gland lesion. The most frequent histological type was adenoma, which occurred in 20 patients (66.7%), followed by hyperplasia in 6 (20%) and atypical adenoma in 4 (13.3%). Of the 2 patients with discordant localization results, case 1 was sporadic PHPT with a single left-sided adenoma. Neck US identified one left-sided nodule (maximum diameter 2.8 cm) and two right-sided nodules (maximum diameter 1.3 cm and 2.3 cm respectively). ^99m^Tc-MIBI scintigraphy indicated a suspected gland on the left side. Bilateral neck exploration was conducted and proved that two right-sided nodules found by US were thyroid nodular goiter. Case 2 was diagnosed as multiple endocrine neoplasia (MEN)1 with an atypical left-sided adenoma. Neck US revealed one left-sided nodule (maximum diameter 2.9 cm) and one right-sided nodule (maximum diameter 2.1 cm). ^99m^Tc-MIBI scintigraphy revealed one left-sided abnormal gland. The patient underwent bilateral neck exploration and single-gland resection on the left side. No other parathyroid gland lesions were found. In summary, the sensitivity of US localization was 100% and the accuracy was 93.8%.

**Fig. 2:**
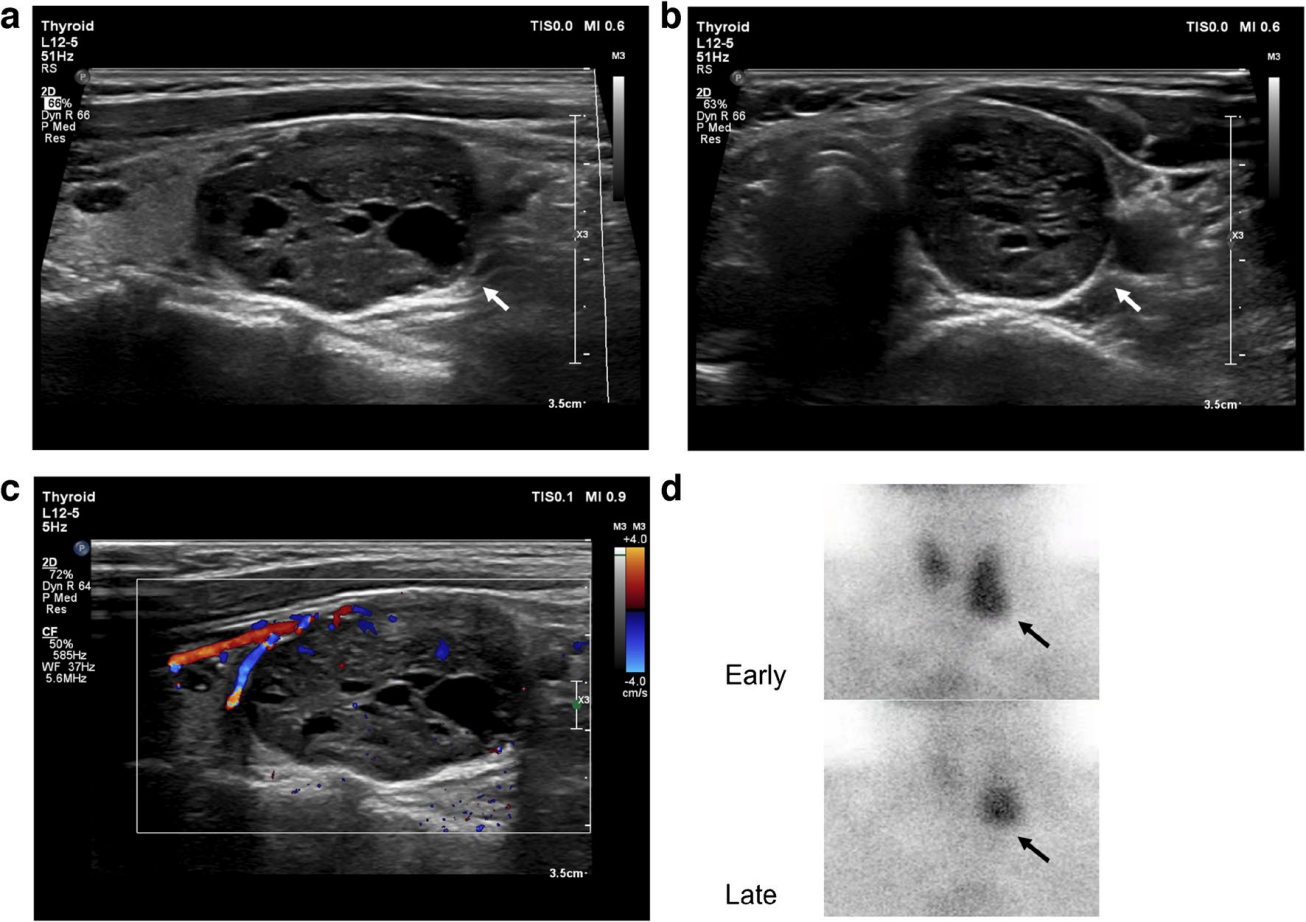
A parathyroid gland adenoma in an 18-year-old girl. **a**, **b** Longitudinal (**a**) and transverse (**b**) ultrasound images show a nodule (*arrows*) with cystic components below the inferior pole of the left lobe of the thyroid gland. **c** Longitudinal Doppler image shows a peripheral feeding vessel with an arc of vascularity and internal blood flow. **d** Technetium-99 m sestamibi scintigraphy shows a focus of intense accumulation at the level of the inferior pole of the left lobe of the thyroid (*arrows*)

**Fig. 3:**
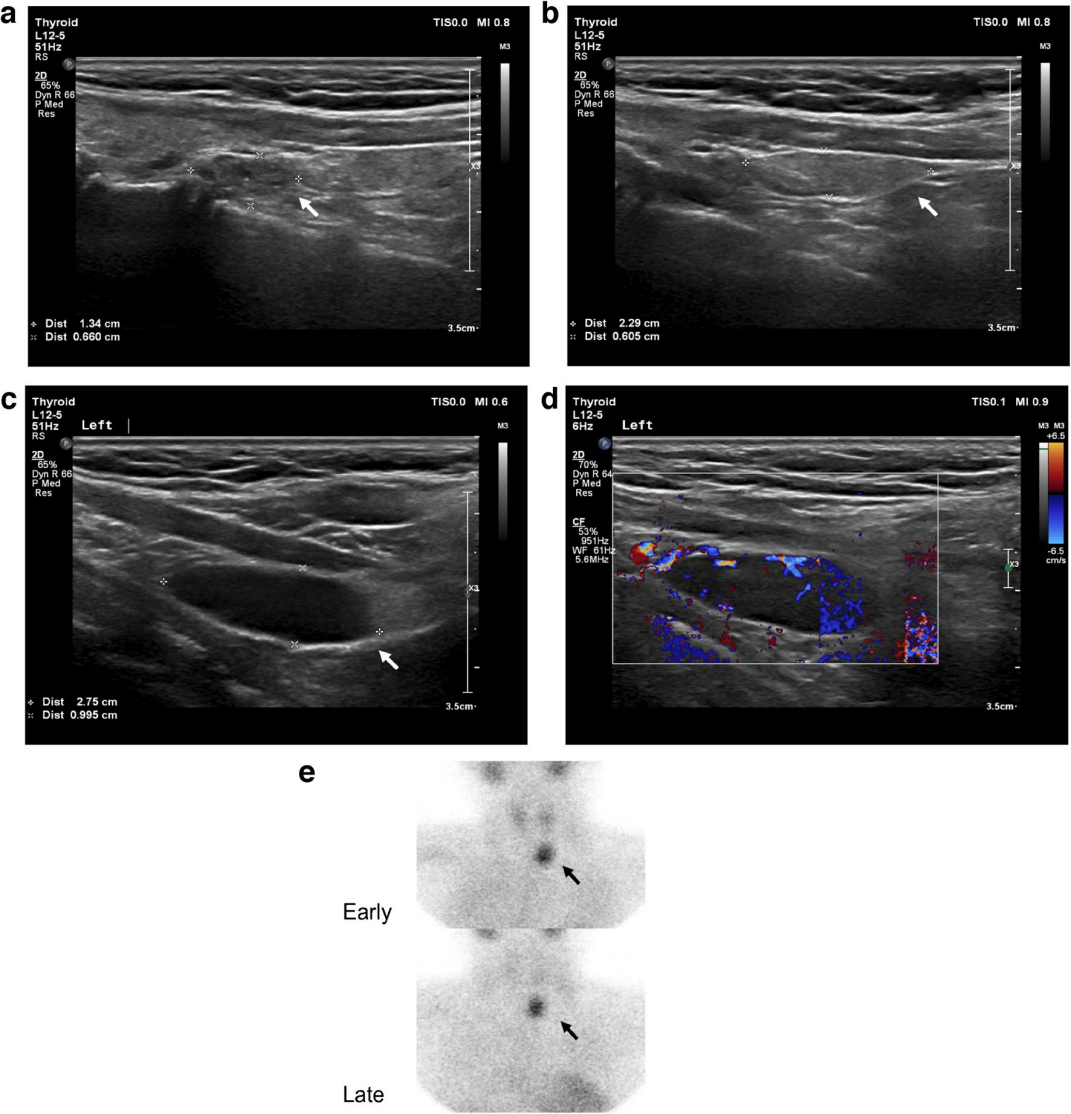
A case of discordant localization between ultrasound (US) and technetium-99 m sestamibi (^99m^Tc-MIBI) scintigraphy in a 13-year-old boy. **a**, **b** Longitudinal US images show two indeterminate nodules (*arrows*) on the right side of the neck without feeding vessels on Doppler mode (images not shown). Histology confirmed a nodular goiter. **c**, **d** Longitudinal US images suggestive of a parathyroid lesion on the left side of the neck. The grayscale image (**c**) shows a hypoechoic oval-shaped nodule with a well-circumscribed margin (*arrow*). The color Doppler flow image (**d**) illustrates a feeding vessel and internal blood flow. Histology confirmed a parathyroid adenoma. **e** ^99m^Tc-MIBI scintigraphy shows a focus of intense accumulation at the level of the inferior pole of the left lobe of the thyroid (*arrows*)

Across the 32 patients, US reported 35 nodules. Among the true positive lesions, 32 (100%) were hypoechoic with oval shapes, circumscribed margins and internal blood flow on color Doppler mode. Cystic changes were found in 4 nodules (12.5%) and a hyperechoic focus was found in 1 nodule (3.1%). Neck US and ^99m^Tc-MIBI scintigraphy images of a parathyroid gland adenoma with concordant localization are shown in Fig. 2. Another case with discordant localization results on US and ^99m^Tc-MIBI scintigraphy is shown in Fig. 3.

## Discussion

PHPT is less prevalent but more severe in pediatric patients than in adults and parathyroidectomy is the only curative therapy. Preoperative localization plays a crucial role in achieving operative success. First-line imaging localization modalities include US and ^99m^Tc-MIBI scintigraphy which have a comparable diagnosed efficacy in adults [8]. A recent study revealed that adding ^99m^Tc-MIBI scintigraphy after positive US results might have limited value since ^99m^Tc-MIBI scintigraphy can only correct the operative plan in 2.3% of patients and the proportion is 0.35% when the lesion size is over 1.2 cm [13]. Ebner et al. revealed a similar operative success in three groups with only US, only ^99m^Tc-MIBI scintigraphy, or US-^99m^Tc-MIBI scintigraphy matched positive results, indicating that one type of imaging examination with positive results might be adequate for preoperative localization [16]. Children and adolescents are a special population and should be exposed to as little radiation exposure as possible. There are limited studies in PHPT imaging analysis focusing on the pediatric population, lacking the evidence to elaborate on the value of a second imaging examination after obtaining a positive US result for children and adolescents. Our study shows that preoperative US localization had a high sensitivity (100%) and accuracy (93.8%) in this pediatric cohort while ^99m^Tc-MIBI scintigraphy only corrected localization in 6.3% patients after positive US results, which is similar to what is already known.

In our study, parathyroid lesions were solitary, large in size and had eutopic localization.

Lesion size can be a contributing factor to imaging localization efficacy. With increases in lesion size, the sensitivity of imaging examinations also increases [17]. Yalon et al. confirmed that the proportion of patients with positive US who received correction by ^99m^Tc-MIBI scintigraphy decreased to 0.35% (1/287) when the lesion size was over 1.2 cm, compared to 2.3% (12/513) in the whole cohort [13]. For the pediatric population, lesion size or weight was quite different among multiple cohorts. The reported mean lesion weight varys from less than 0.4 g to more than 5 g [18–20]. In our group, the median lesion maximum diameter of parathyroid gland lesions was 2.85 cm (range 1.0–5.8 cm) and 75% were over 2 cm. This could help explain the outstanding performance of US.

Multigland disease is one of the most difficult situations for preoperative localization imaging. It is widely accepted that there is an association between multigland disease and MEN1 in the pediatric population. MEN1 patients may have have a higher incidence of multigland disease [3, 21]. In our study, 5 (15.6%) patients were diagnosed with MEN1 and no multigland disease was found, nevertheless 3 in 5 MEN1 patients had recurrent lesions after a follow-up of 1–7 years. To our knowledge, sufficient evidence that the combination of US and ^99m^Tc-MIBI scintigraphy could increase the sensitivity and specificity compared to single US in an underage population with multigland disease is lacking and further study might be needed.

Ectopic glands are another challenge for localization [10]. The proportion of ectopic adenomas in adult patients is 6–16% [22]. In two studies with 52 (<19 years old) and 86 (<22 years old) patients, ectopic lesions accounted for 9.6% and 25%, respectively [22, 23]. In our group, no ectopic location was reported according to surgical findings. Thus, a larger cohort might be needed for further study.

The above features of our cohort might explain the excellent performance of US. It would be interesting to include PHPT patients with negative US and further discuss the performance of ^99m^Tc-MIBI scintigraphy in such patients in future studies.

There are several limitations of this study. First, the PHPT patients were enrolled from a national tertiary care referral center; thus, they represented more severe conditions which might lead to selection bias. Second, since this is a retrospective study, it was difficult for radiologists to be completely blinded to other imaging examinations before reaching a diagnosis. A future prospective study is warranted. Third, although our cohort is one of the largest pediatric PHPT cohorts focusing on imaging analysis, we only enrolled 32 patients. Sampling error might be the possible reason why this study has a higher percentage in which MIBI would dictate the surgical approach compared to an adult PHPT cohort [13].

## Conclusion

In our cohort, parathyroid lesions of pediatric PHPT patients were solitary, large in size and had eutopic localization. Neck US has high sensitivity for identifying abnormal glands and is useful for preoperative localization, while adding ^99m^Tc-MIBI scintigraphy for those with positive US results would not improve the sensitivity. Implementation of ^99m^Tc-MIBI scintigraphy might increase the specificity in pediatric patients with suspected multigland disease as shown on US.

## Data Availability

The data generated during the current study are available from the corresponding author on reasonable request.
